# Treatment of Visceral Leishmaniasis

**DOI:** 10.4103/0974-777X.62883

**Published:** 2010

**Authors:** E M Moore, D N Lockwood

**Affiliations:** 1 *Hospital for Tropical Diseases, University College London Hospital*; 2 *London School of Hygiene & Tropical Medicine*

**Keywords:** Ambisome, Amphotericin, Antimony, Miltefosine, Paromomycin, Pentamidine, Visceral leishmaniasis

## Abstract

The available treatment options for visceral leishmaniasis (VL) have problems relating to efficacy, adverse effects and cost, making treatment a complex issue. We review the evidence relating to the different methods of treatment in relation to - efficacy and toxicity of the drugs in different areas of the world; ability to monitor side effects, length of treatment; ability of patients to pay for and stay safe during treatment, ability of the healthcare services to give intramuscular, intravenous or oral therapy; the sex and child-bearing potential of the patient and the immune status of the patient. The high mortality of untreated/ poorly treated VL infection makes the decisions paramount, but a unified and coordinated response by each area is likely to be more effective and informative to future policies than an *ad hoc* response. For patients in resource-rich countries, liposomal amphotericin B appears to be the optimal treatment. In South Asia, miltefosine is being used; the combination of single dose liposomal amphotericin B and short course miltefosine looks encouraging but has the problem of potential reproductive toxicities in females. In Africa, the evidence to switch from SSG is not yet compelling. The need to monitor and plan for evolving drug failure, secondary to leishmania parasite resistance, is paramount. With a few drugs the options may be limited; however, we await key ongoing trials in both Africa and India to explore the effects of combination treatment. If safe and reliable combinations are revealed by the ongoing studies, it is far from clear as to whether this will avoid leishmania parasite resistance. The development of new drugs to add to the armamentarium is paramount. Lessons can be learnt from the management of diseases such as tuberculosis and malaria in terms of planning the switch to combination treatment. As important as establishing the best choice for specific antileishmanial agent is ensuring treatment centers, which can best manage the problems encountered during treatment, specifically malnutrition, bleeding, intercurrent infections, drug side effects and detecting and treating underlying immunosuppression.

## INTRODUCTION

There are an estimated 500,000 new cases per year of visceral leishmaniasis (VL) globally.[1] Although 90% of the new cases occur in just five countries (India, Bangladesh, Brazil, Nepal and Sudan), the unique problems posed by the disease in each setting affect the choice of treatment. In South Asia and East Africa, humans with VL, or post *kala-azar* dermal leishmaniasis, are the main reservoir for ongoing transmission of infection. Therefore, partially treated patients from these areas can develop VL parasites resistant to treatment, which in turn may be transmitted to new patients causing ‘primary drug resistance’ as has happened in India. In other foci, such as the Mediterranean, Middle East and Brazil, where the domestic dog is the principle reservoir of infection, parasite drug resistance is not such a concern. However, the infection principally occurs in children or immunocompromised adults in these areas, which also affects treatment choices. Often, in East Africa, treatment is given under difficult field conditions with little possibility of monitoring or follow-up to malnourished and under-the-threat-of-war population. The cost of treatment is important when patients need to pay for treatment. In India, the healthcare provision for VL patients is heterogeneous with a poorly standardized system of private care, which may entail great expense to the patient's relatives. Treatment choice is therefore affected by the patient's financial status. In East Africa, much of the treatment is given free by non-governmental organizations. The strength of evidence for various therapies, including treatment length and dosing regimens, vary, and there may be fundamental differences in the pathogen behavior and the host response to the pathogen in the different settings.

In reviewing the treatments for VL in this paper, we will not simply highlight the different drugs used, but their use in different environments in terms of efficacy, cost, and feasibility of safe administration in difficult environments and the impact of HIV co-infection. We also highlight the adjunctive care that is important in treating VL patients.

### Treatment

The main drugs available for treatment of VL are the systemic agents like antimony, amphotericin, paromomycin and now the oral drug miltefosine. All these drugs will be discussed in detail and the evidence for their use in particular settings noted.

#### A) Choice of antileishmania agent

Table 1 summarizes the different agents used to treat VL patients, and their principle use.

**Table 1:** Summary of VL treatment options

VL therapy	Advantages	Disadvantages	Places used in
Sodium stibogluconate (SSG)	Long history of effective treatment (even in difficult circumstances)	Toxicities (vomiting, cardiac, liver)	East Africa
	Cheap generic preparations available	Clinical treatment failure in India	
		Lengthy treatment	
		No oral preparation	
Amphotericin B	Good efficacy, especially in those with treatment failure with SSG	Toxicities (infusion reactions, renal)	India
		Lengthy treatment	
		No oral preparation	
Liposomal amphotericin B	Excellent efficacy, even for HIV patients	Expensive	Resource rich countries
	Short treatment course	No oral preparation	
Miltefosine	Oral preparation	Reproductive toxicity	India
	Good efficacy in patients without HIV	Toxicities (gastrointestinal) ?poorer efficacy (but lower mortality) compared to SSG in HIV patients	
Paromomycin	Cheap	Variable drug supply	India
	Equivalent cure rates to Amphoterecin B in India (probably not in Africa)	Toxicities (ototoxicity, liver)	In combination in
	Broad spectrum activity thus helpful in intercurrent diarrheal illnesses in African cohorts	No oral preparation	Africa with SSG
Pentamidine	Useful for secondary prevention in HIV patients	Poor efficacy as primary treatment course	South America
		Toxicities (cardiac, diabetes, gastrointestinal)	Prophylaxis for HIV patients in Europe

##### Antimony based drugs

The heavy metal, antimony, has been the basis of drug treatment for VL since the 1940s. Currently, there are two formulations in use: sodium stibogluconate (SSG), which contains 100mg antimony/100ml ‘Pentostam’- Glaxo-Smith-Kline, generic sodium stibogluconate - Albert David Company and meglumine antimoniate, which contains 85 mg antimony/100 ml (‘Glucontime’- Rhone-Poulenc). Both formulations have comparable efficacy and toxicity.[2] They have poor oral absorption and are given via intramuscular or intravenous injections. In the 1980s, dosage studies clarified that a dosage of 20 mg/kg/day rather than 10 mg/kg/day improved the cure rate with no substantial increase in toxicities. The treatment course should last at least 20 days, preferably 28 days.[3,4] Common side effects include nausea and vomiting, arthralgia, hepatitis, pancreatitis and cardiac dysrhythmias. A detailed review of side effects of SSG when used to treat otherwise healthy patients for cutaneous or mucocutaneous leishmaniasis, with frequent monitoring for toxicities, found that 67% of patients developed a raised serum amylase level whilst 85% developed abnormal serum alanine transaminase levels, most frequently in the third week of treatment, indicating a cumulative toxicity. However, despite the biochemical abnormalities seen, severe clinical adverse events were unusual and rarely led to treatment stoppage;[5] 10% of this same cohort developed a prolonged QTc interval on electrocardiogram (ECG).

Despite the side effects of SSG, and the need to monitor for toxicities, it has been used successfully in field sites with minimal monitoring. The aid organization ‘Medicine-Sans-Frontiers’ (MSF) have treated huge numbers in Southern Sudan since the epidemic of the 1990s in a malnourished war-torn population.[6] Seaman *et al.* describe treating 3076 patients in 1990 in one treatment center in Sudan with 30 days of SSG (20 mg/kg) and achieved an 83.3% cure rate with 3% relapse, and 10.9% death rate. Death was associated with markers of disease severity (extremes of age, a history of prolonged illness prior to treatment, severe anemia, a low body mass index, a large spleen size and a high parasite load on splenic aspiration) but also with the presence of vomiting. Little monitoring was available to determine how many of the deaths were associated with toxicities, but as a mass treatment in difficult circumstances, the outcomes are encouraging. Gastrointestinal symptoms appear a major risk factor for death during treatment with SSG from subsequent studies from the MSF teams in East Africa; a fifth of the 3365 treated by MSF in Southern Sudan from 1998-2002 experienced diarrhea and/or vomiting, leading to a 3.1 times increased risk of death during treatment.[7]

Studies from India have focused on the potential cardiac toxicities of SSG. A cohort of 80 VL patients treated with SSG in Bihar state, India, developed a range of ECG abnormalities; prolonged QTc interval (8%), ST elevation (4%), T wave inversion 9% and arrhythmias (6%).[8]

Clinical resistance to SSG has been a problem in India since the late 1990s. The toxicity study described above had a cure rate of 60%.[8] Later Sundar *et al.* described the geographical distribution of the reported resistance to SSG with 35% long-term cure rate in Bihar state compared to 86% in Uttar Pradesh.[9] Reports from Nepal have revealed a lower cure rate in the district next to Bihar state of 76% compared to 90% in other districts in Nepal.[10] By 2005 the magnitude of the problem was revealed by studies in Bihar with an initial end of treatment failure rate of 43%, but a further 27% of patients having relapsed within six months of the end of treatment.[11] Potential reasons for the development of VL parasites resistant to SSG in Bihar include incomplete courses of SSG (as patients could not afford the full length course) and use of cheaper generic preparations of SSG which were initially poorly monitored for drug quality and consistency. Subsequently, however, better drug quality control measures have led to successful generic SSG use in East Africa.[12,13] The anthroponotic nature of the VL epidemiology in India, with man as the main reservoir for new infections, clearly contributed to the development of drug resistant parasites in Bihar state.

The clinical failure of SSG in India has raised concerns for the other foci with similar anthroponotic epidemiology in East Africa. So far there has been little evidence for clinical failure with SSG use in Africa, although in Sudan the 2.7% clinical failure in patients treated with SSG did correlate with *in vitro* parasite resistance to SSG.[14] Data from MSF treatment centers in Ethiopia, in 2006, treating HIV negative VL patients with SSG report cure rates of 95% initial cure, 1% initial clinical failure, and 77% confirmed as a final cure at six months.[15] This data also highlighted the impact of HIV co-infection on cure rates; patients in the same cohort co-infected with HIV had a 90% initial cure rate, 1% initial failure, but a 57% final cure rate. HIV and VL have a complex interaction and definitive cure has been difficult to achieve with any treatment in the presence of HIV infection. The toxicities of SSG may also be worsened by the presence of HIV infection. A study from Spain reported ‘adverse events’ in 2.5% of HIV negative patients treated with SSG compared to 18.5% in those with HIV.[16] However, adverse events were defined as renal toxicity, anemia or a raised serum amylase greater than twice the upper limit of normal. Clinically adverse events were not reported, and some of the biochemical changes might be due to HIV infection *per se*, rather than the SSG. The markedly different mortality rates in patients treated with SSG in Ethiopia with and without HIV (HIV positive Mortality = 33.6% vs. HIV negative Mortality = 3.6% during SSG treatment course) may in part be from SSG toxicities. However, there was a non significant difference in the occurrence of vomiting in those with and without HIV in this cohort (HIV pos. = 44.4% vs. HIV neg. =35.7%). The evidence is therefore not strong enough to say conclusively that SSG toxicities worsen in HIV co-infection.

##### Amphotericin B and Liposomal Amphotericin B

Although amphotericin B (AB) has excellent *in vitro* activity against Leishmania parasites, its use was initially limited by toxicities. Liposomal Amphotericin B (LAB) has better tissue penetration and is more effective at lower doses, reducing toxicities.[17,18] A study from Bihar in 2004 compared clinical efficacy of Amphotericin B (1 mg/kg alternate days for 30 days) and Liposomal Amphotericin B (2 mg/kg for five days) for treating patients with VL and found similar high definitive cure rates at six months (96% cure in both groups).[19] However, drug infusion reactions characterized by fever and/or rigors were very common in the AB groups (98% experienced a reaction, with 8.4 mean number of reactions per patient) as compared to the LAB group with 71% having no such reactions. Other adverse reactions, such as hypokalaemia, renal dysfunction and a fall in hemoglobin levels were significantly more common in the AB group too. There were notable differences in costs for the treatment courses with the AB course costing $ 417 per patient whereas the LAB course costing $ 872 per person. The expense of LAB has therefore limited its use in many areas despite better side effect profile.

The use of LAB was initially limited in the early 1990s in resource rich countries to a second line drug when VL parasites persisted despite SSG treatment, particularly in the HIV-infected patients.[20] In the mid 1990s, the MSF teams in Southern Sudan were beginning to use LAB in difficult field sites to treat patients with severe disease with excellent effect.[21] A key multicenter European trial in 1996 showed that short course LAB (20 mg/kg in five doses over 10 days) had excellent cure rates and low toxicities.[22,23] This regimen has now become the standard for resource rich countries in patients with normal immune systems.

The expense of LAB, for treatment of VL, initially limited its use in India despite there being clinical evidence of parasite resistance to SSG. However, a direct comparative study of SSG and AB showed better outcomes with AB, despite its inferiority to LAB in terms of toxicities, with 70% definitive cure rates in SSG group compared to 100% cured in the AB group.[24] Much of the research since, particularly from India, has been to reduce the course of LAB whilst retaining effectiveness, to limit costs to patients. A randomized dose finding study performed on 30 patients in Bihar found similar excellent cure rates in patients given a total dose of 14 mg/kg LAB over 10 days as those given total dose 6 mg/kg of LAB over 10 days.[25] Another study from Bihar on 91 patients comparing LAB at 5 mg/kg single dose or split into five doses over five days found equivocal cure.[26] Subsequently, a multicenter study of 203 patients, in Bihar, given a single dose of LAB at 7.5 mg/kg achieved 96% initial cure and 90% six-month cure with no increase in toxicities.[27] Most recently, a Phase III study in India[28] compared a single infusion of LAB in a dose of 10 mg/kg body weight in 304 patients with VL, with conventional treatment of 14 infusions of AB, in a dose of 1 mg/kg body weight on alternate days for 14 doses to 108 patients with VL. This single-dose treatment regimen could revolutionize the approach to the control of visceral leishmaniasis through simplification of management, reduction of costs of drugs and hospitalization, greater safety and tolerance for patients, and better convenience and economy for their families.

Clinical failure to LAB has rarely been observed. A study from Sudan of 64 patients treated with a total dose 15-49 mg/kg of LAB for complicated or relapsed VL found 16% with a clinical treatment failure with persistent parasites in the lymph nodes at the end of the treatment. Although 40% of these patients had severe underlying disease (HIV or TB) the most notable feature was the initial high parasite density at diagnosis. Therefore this situation is likely to represent inadequate treatment with LAB rather than true parasite resistance to the drug.[29] Furthermore, the mode of action of AB, on membrane ergosterol, is such that an organism would have to undergo fundamental changes in order to become resistant. All Indian patients who have relapsed after treatment with single-dose L-AmB have been cured with a five-day regimen of L-AmB. Therefore parasite resistance is considered unlikely to develop in response to monotherapy with L-AmB, especially single dose monotherapy.

However, low doses of LAB used with success in India might not translate to the Africa setting where parasite load appears to be higher. Currently, the WHO has agreed a preferential price with the manufacturer (Gilead) for distribution of LAB for health programs in developing countries, but it remains more expensive than other first line regimes. A cold chain is also required for LAB, as high or low temps (< 4° or > 25°C) may alter the liposomal characteristics, theoretically affecting toxicity and efficacy of the drug.[17]

A WHO working group made the following recommendations for use of LAB in 2005:[30]

1. In the zoonotic foci of the Mediterranean, Middle East and Brazil – a total dose of LAB of 20 mg/kg with variable dosing regimes.
2. In anthroponotic foci of South Asia and Horn of Africa – when unresponsiveness to antimony exceeds a threshold (to be decided by each region) consider an alternative first line, either an amphotericin B formulation or a combination treatment.

##### Miltefosine

Miltefosine is a membrane active alkyl phospholipid, originally used as an anticancer drug. It was found to have antileishmania activity in animal models in the early 1990s.[31,32] It is active orally and early studies suggest that the optimal dose to balance efficacy and tolerance be 100 mg/day for 28 days.[33] Animal studies have shown that it has some reproductive toxicity and thus it is contraindicated in pregnancy, and needs to be used with caution in women of reproductive age. Gastrointestinal side effects appear to be common but rarely severe enough to warrant stopping treatment.[34] In India, a Phase 3 trial comparing miltefosine with AB found similar cure rates at six months (miltefosine-94% vs. AB-97%), but more vomiting in patients taking miltefosine (Miltefosine-38% vs. AB-20%). However, AB infusion reactions were very common (90%) and renal impairment was more common in patients treated with AB (Milt-16% vs. AB-60%).[35] A study from Ethiopia treating VL patients of whom 29% were co-infected with HIV, compared miltefosine with SSG. Initial cure, mortality and initial treatment failure rates were equivocal in patients without HIV co-infection. However, in HIV/VL co-infected patients miltefosine appeared to be safer (mortality miltefosine = 6% vs. SSG = 12%) but was less effective than SSG (initial treatment failure miltefosine-18% vs. SSG-10%).[15] A large Phase 4 study from India has reported encouraging results of treating VL as an outpatient with miltefosine.[36] Of the 1132 patients, the final cure rate at six months was 82% which is similar to cure rates from local hospital care. Only 3% of patients had Grade 3 toxicities, confirming that miltefosine can be used as an outpatient treatment. However, the long half life (seven days) makes the risk of developing parasites resistant to miltefosine a real possibility.

in the *Kala-azar* Elimination Program, through which India, Nepal and Bangladesh introduce treatment with miltefosine in a phased manner, is now being used in 10 Indian districts. It will be expanded to almost the whole of Nepal, but has not yet been implemented in Bangladesh. Second line treatment is AB.

##### Paromomycin

Paromomycin (Aminosidine) is an aminoglycoside with a broad spectrum of activity against gram negative bacteria, some gram positive bacteria, mycobacteria, some cestodes and leishmania parasites. It must be given by intramuscular injections and the main side effects include ototoxicity, injection site pain and raised liver enzymes.

Drug development has been slow despite initial encouraging evidence from proof of concept studies in the early 1990s[37] because of insecure drug supplies from manufacturers.[38] In the mid 1990s, paromomycin was promoted as an alternative treatment to SSG because of the evolving clinical failure of SSG in India. MSF have ensured continued drug supply through various manufacturers during this time as it has proved very useful in the overcrowded VL treatment camps in the epidemic foci in Southern Sudan. The high mortality during VL treatment in these areas was thought to be due in part to outbreaks of concomitant diarrheal illness. Seaman *et al.* compared a shorter course of SSG (17 days) in combination with paromomycin to the conventional 30-day SSG regimen for patients in Sudan and found equivalent efficacy but lower mortality rates in the short course paromomycin/SSG group.[39] Since then this regimen has been used by MSF in similar situations with good outcomes.[40] In India, direct comparative studies of paromomycin (dose 16 mg or 20 mg/kg for 21 days) and SSG (standard course) showed a higher cure rate for patients receiving paromomycin.[41] A key Phase 3 study by Sundar *et al.* in India in 2007 compared AB (dose-1 mg/kg alternate days for 30 days) and paromomycin (dose-11 mg/kg for 21 days) and showed similar six-month cure rates (paromomycin = 94.6% vs. AB = 98.8%) which subsequently led to licensing for its use in India.[42] Patients treated with paromomycin had a 6% adverse event rate (raised liver enzymes, ototoxicity and injection site pain) compared to 2% in patients treated with AB (renal dysfunction and fever/rigors). Shortening the course of paromomycin from 21 to 14 days has subsequently shown inferior cure rates in India.[43] It is the cheapest treatment for VL, costing E4.19 for a 21-day course for a 35 kg man. *In vitro* resistance of leishmania parasites to paromomycin has been demonstrated readily, but this is not yet a significant clinical problem. So there have been calls for its use in combination only to avoid future problems with clinical failure.[38] An ongoing multicenter study from East Africa shows that the dose of 11 mg/kg of paromomycin successfully used in India is not sufficient for effective cure in Africa, and factors relating to the host or parasite that may affect drug efficacy. The dose has been increased to 20 mg/kg and results are awaited.[44]

##### Pentamidine

Pentamidine was once the preferred second line drug in cases of SSG resistance, but studies have shown inferior cure rates to amphotericin B.[45] It has well known toxicities (cardiac, diabetes mellitus, hypotension, gastrointestinal side effects) that have limited use. However, it appears to be useful in prevention of relapse in patients with successful initial cure with another agent but have a high risk of relapse secondary to HIV and other immunodeficiency.[46]

#### New agents – Sitamaquine

Sitamaquine is an 8-aminoquinolone analogue of Primaquine. Phase 2 studies have been conducted in a range of VL settings with variable results. In Kenya, used at a dose of 1 mg/kg for 28 days, it had a 50% final cure rate.[47] In Brazil, the dose of 2 mg/kg had a 67% cure rate but unexpected nephrotoxicities in higher doses.[48] In India, doses of 2-2.5 mg/kg have reported 80-100% cure rate with common toxicities of vomiting, dyspepsia, nephrotoxicity and cyanosis secondary to methaemoglobinaemia.[49] Further dose finding studies in Kenya using 1.75-3 mg/kg had 80-90% cure rate; abdominal pain and headache were common but tolerable side effects, and rare but severe nephrotoxicity were seen at the higher doses.[50]

#### Combination treatment

Recently, the role of combination treatment has been discussed as a way of preventing the leishmania parasites developing resistance to chemotherapeutic agents.[51,52] The alarming growth of SSG clinical failure in India has fueled this debate. There are a few therapeutic options for the treatment of VL, relatively little research into newer agents, and a high mortality rate if treatment does fail. There are pharmokinetic reasons as to why leishmania parasites develop easy resistance to the newer agents of miltefosine and paromomycin. The main anthroponotic foci in India and Africa provide a perfect ground for evolving primary drug resistance. The difficulties in achieving definitive cure in HIV/VL co-infected patients adds to the situation as these patients harbor drug exposed parasites which may be transmitted on to others. There is also a desire to give the shortest treatment course possible to achieve cure which may well be better achieved by combination treatment. This might avoid excessive costs for patients (especially in South Asia settings) and would be helpful in VL foci unsafe from war (Southern Sudan). However, this approach has not been fully validated and there are risks of augmenting drug toxicities, which in many areas are difficult to monitor for and manage. VL patients who are co-infected with HIV now have greater access to effective antiretroviral treatment and thus there are risks that these patients may be taking several drugs at the same time leading to greater risk of drug interactions and toxicities and therefore potentially compliance issues. There is also evidence that there may be some cross resistance between the various agents discussed above,[53] which may mean that even with combination therapy, infections are difficult to cure. The combination of SSG and paromomycin as described earlier appears to have been an effective practical approach used by MSF for many years in Africa.[39] Sundar *et al.* have shown good results with single-dose ambisome followed by 7-14 days of miltefosine[54] in India, although there is no data to support the contention that this regimen will avoid the development of parasite resistance to miltefosine. Trials are ongoing in Africa and India evaluating various drug combinations.[55]

### B) Adjunctive treatments

Specific antileishmania agents are only part of the treatment for VL patients. Supportive care includes managing and preventing the complications of VL. Severe wasting is a dominant feature of VL patients and therefore dietary support has become standard in many VL treatment programs, particularly in East Africa where there may be concurrent food insecurity. MSF VL treatment programs emphasize the importance of high calorific supplementation and vitamin supplementation (multivitamins, vitamin C and A). The spleen in VL patients, although large, is probably dysfunctional due to the infiltration with leishmania parasites, and thus the risk of developing malaria in endemic areas is greater. Concurrent malaria is also likely to be of greater severity due to the anemia and thrombocytopaenia from leishmania parasite infiltration of the bone marrow. The use of prophylactic antimalarials during VL treatment and bed nets has become standard in many treatment centers in East Africa. The use of bed nets may also help with ongoing transmission of VL, although sandflies are much smaller than mosquitoes and therefore a fine mesh net is required. Folate and iron supplementation can also be given to aid recovery from anemia during VL treatment. Intercurrent infections are problematic during VL treatment whilst bone marrow function is poor, particularly from gut pathogens. Some VL treatment centers use weekly prophylactic antibiotics to prevent this. The reduction in mortality seen when using paromomycin in addition to SSG treatment in Sudan[39] may be in part from the antibacterial effects of paromomycin. Simple measures such as ensuring a clean water supply at the treatment centers are as important in preventing the high mortality from gut pathogens during treatment. Prompt recognition and management of intercurrent infections is vital to prevent high mortality during VL treatment and this requires staff training and essential drug availability. Bleeding problems from thrombocytopaenia, particularly epistaxis, is another complication seen frequently in VL with high mortality if the equipment and trained staff are not available to manage this complication effectively. Some VL treatment centers in East Africa give Vitamin K to those at high risk (pregnant women, patients with severe VL or very low hemoglobin), or patients actively bleeding. Side effects from the specific antileishmania agents discussed above need to be monitored for. In resource rich countries this may involve frequent analysis of blood biochemistry and hematology and ECGs, but must not be forgotten in resource poor countries where simple measures to effectively manage symptoms such as nausea and vomiting should be undertaken. The difficulties involved with achieving definitive VL cure when patients are co-infected with HIV have been described above. An essential part of management of VL patients is to establish their HIV status and give effective antiretroviral treatment if required. At the end of treatment, it is essential to assess whether the VL treatment has been successful. Performing an aspirate from lymph nodes or spleen, to assess parasite load, is a good objective measure but must be done with clinical measures of disease improvement such as absence of fever, weight gain and spleen size regression.

## CONCLUSION

VL remains a challenging disease to treat. We have reviewed the efficacy and safety of antimony. Newer drugs such as liposomal amphotericin are promising, especially in the single day treatment regimen but need to be tested in different leishmania endemic settings. The experience with paromomycin, where a drug effective in India was not effective in East Africa shows that there are multiple factors to be considered in the development of an effective leishmania treatment program. Miltefosine is promising as an oral drug. It is very important that the few new drugs that are available are protected and not used in ways that would enhance the development of resistance.
